# Meta-analysis of executive functioning in ecstasy/polydrug users

**DOI:** 10.1017/S0033291716000258

**Published:** 2016-03-11

**Authors:** C. A. Roberts, A. Jones, C. Montgomery

**Affiliations:** 1 Department of Psychological Sciences, University of Liverpool, Liverpool,UK; 2 School of Natural Sciences and Psychology, Liverpool John Moores University, Liverpool,UK

**Keywords:** Ecstasy, executive function, meta-analyses, 3,4-methylenedioxymethamphetamine

## Abstract

Ecstasy/3,4-methylenedioxymethamphetamine (MDMA) use is proposed to cause damage to serotonergic (5-HT) axons in humans. Therefore, users should show deficits in cognitive processes that rely on serotonin-rich, prefrontal areas of the brain. However, there is inconsistency in findings to support this hypothesis. The aim of the current study was to examine deficits in executive functioning in ecstasy users compared with controls using meta-analysis. We identified *k* = 39 studies, contributing 89 effect sizes, investigating executive functioning in ecstasy users and polydrug-using controls. We compared function-specific task performance in 1221 current ecstasy users and 1242 drug-using controls, from tasks tapping the executive functions – updating, switching, inhibition and access to long-term memory. The significant main effect demonstrated overall executive dysfunction in ecstasy users [standardized mean difference (SMD) = −0.18, 95% confidence interval (CI) −0.26 to −0.11, *Z* = 5.05, *p* < 0.001, *I*^2^ = 82%], with a significant subgroup effect (*χ*^2^ = 22.06, degrees of freedom = 3, *p* < 0.001, *I*^2^ = 86.4%) demonstrating differential effects across executive functions. Ecstasy users showed significant performance deficits in access (SMD = −0.33, 95% CI −0.46 to −0.19, *Z* = 4.72, *p <* 0.001, *I*^2^ = 74%), switching (SMD = −0.19, 95% CI −0.36 to −0.02, *Z* = 2.16, *p <* 0.05, *I*^2^ = 85%) and updating (SMD = −0.26, 95% CI −0.37 to −0.15, *Z* = 4.49, *p <* 0.001, *I*^2^ = 82%). No differences were observed in inhibitory control. We conclude that this is the most comprehensive analysis of executive function in ecstasy users to date and provides a behavioural correlate of potential serotonergic neurotoxicity.

## Introduction

Ecstasy (3,4-methylenedioxymethamphetamine; MDMA) remains popular despite reports of potential long-term negative consequences associated with repeated use (see Parrott, 2013*a*, *b*). Furthermore, ecstasy poses a major public health concern due to an increase in recent MDMA-related deaths (Anderson, 2014) as well as reported increases in tablet strength, with some sources suggesting tablets may contain upwards of 200 mg of MDMA (Global Drugs Survey; Winstock, 2015). Animal literature suggests that ecstasy causes damage to serotonin axons (Ricaurte *et al*. 1988; Molliver *et al*. 1990). There is also evidence of ecstasy-related alterations in mood (Curran *et al.* 2004) and long-term changes in neuroendocrine function (Wetherell & Montgomery, 2014). However, perhaps public health warnings are not being taken seriously due to mixed messages in the media and scientific literature about relative harms of drugs (for assessment of drug-related harms, which poorly correlate with UK drug classification, see Nutt *et al.* 2010).

A recent review by Murphy *et al.* (2009) suggests that ecstasy-related cognitive dysfunction is not consistently reported in the literature, thus monitoring of research is necessary to gain a coherent understanding of drug effects. Executive functions (EFs) have been defined as a set of general-purpose control processes, required for regulating thought and action (Miyake & Friedman, 2012). Moreover, the central executive is an integral component of working memory (Baddeley, 2000) and is required for coordinating and processing information. Some of the apparent inconsistency in the literature may be attributable to several of the classic working memory/‘executive’ tasks requiring use of multiple EFs: a problem of task impurity (Miyake & Friedman, 2012). An influential EF framework suggested that the central executive is not a unified construct; rather it is comprised of several correlated but distinctly separable functions (Miyake *et al.* 2000). Three discrete EFs were originally identified: mental set shifting/switching (‘switching’); information updating and monitoring (‘updating’); and inhibition of prepotent responses (‘inhibition’). A fourth component, ‘access’ to semantic memory, was later added by Fisk & Sharp (2004). These are the four classic EFs that have been assessed in the literature. However it is interesting to note that more recent developments in the unity/diversity framework (Miyake & Friedman, 2012) suggest that inhibitory control no longer exists as an EF, as it is subsumed under the concept of working memory and EF in general.

Montgomery *et al.* (2005*a*) suggested that there may be a differential pattern of executive impairment based on previous drug use and type of function, whereby ecstasy-related deficits were apparent in updating and access, but not in switching or inhibition. These conclusions were arrived at by administering tasks that are understood to assess one function only. As such, it may be that ecstasy users are impaired on some EFs and not others, supporting the unity and diversity framework (Miyake *et al.* 2000; Miyake & Friedman, 2012). There are nuances in the neuroanatomy underpinning each function, which may explain why impairment is potentially function specific. For example, the dorsolateral prefrontal cortex (DLPFC) is understood to be important for memory updating (Goldman-Rakic, 1996), whereas lesion studies suggest that the left DLPFC in particular is important for letter-based word fluency (Stuss *et al.* 1998). Ability to switch mental set is impaired following damage to the PFC and basal ganglia (Ravizza & Ciranni, 2002), and finally response inhibition performance has long been localized to the PFC; however, of particular importance is the right inferior frontal gyrus (Chambers *et al.* 2009). The conclusions reached by Montgomery *et al.* (2005*a*) and the review by Murphy *et al*. (2009) are that ecstasy use has a stronger detrimental effect on updating and access, and that inhibitory control and mental set switching are unaffected by use. However, there are instances of ecstasy users showing no apparent deficit in function-specific tasks that tap updating (Hanson & Luciana, 2004; Hoshi *et al.* 2007) and access (Gouzoulis-Mayfrank *et al.* 2000; Bedi & Redman, 2008) as well as instances of ecstasy-related impairments in switching (von Geusau *et al.* 2004; Dafters, 2006*a*) and inhibition (Yip & Lee, 2005).

Several neuroimaging studies have concluded that ecstasy-related neuronal adaptations may occur neurophysiologically before they manifest functionally. Roberts & Montgomery (2015*a*) suggested that ecstasy users display increased blood flow to areas of the PFC during a verbal fluency task, despite no differences in task performance. This suggests that ecstasy users work harder to achieve similar performance to controls, and that functional differences may be apparent with increased workload. Similar conclusions have been drawn from electroencephalogram studies whereby ecstasy users display evidence of recruiting additional resources in comparison with controls, whilst showing similar performance (Burgess *et al.* 2011; Roberts *et al.* 2013*a*, *b*, *c*). Similarly, functional magnetic resonance imaging (fMRI) studies have shown alterations to neuronal activation consistent with ecstasy-related damage despite not showing any performance deficits (Moeller *et al.* 2004; Daumann *et al.* 2005; Jager *et al.* 2008; Roberts & Garavan, 2010). Such neuroimaging studies suggest that neurophysiological correlates of executive performance are present before a behavioural difference manifests itself. It remains plausible that many behavioural studies lack statistical power to observe subtle impairments over the entire spectrum of EFs. Therefore, the aim of this meta-analysis was to examine the evidence for overall dysfunction of executive control in ecstasy users compared with polydrug users, but also to examine any functional specific deficits.

## Method

### Eligibility criteria

#### Participants

Included studies were those assessing EF in human ecstasy/MDMA users aged 18 years+, who did not have a history of major psychiatric or neurological problems. Ecstasy user groups were eligible if they were described as current ecstasy users; control groups were eligible if they reported some use of drugs, but no ecstasy use – with the exception of studies in which the ecstasy users were recruited with the specific criteria of limited exposure to other drugs. In each case, participants were not intoxicated at the time of testing. The majority of studies included used a minimum abstinence period of 7 days, with the exception of Heffernan *et al.* (2001), de Sola *et al.* (2008*a*) and Fagundo *et al.* (2010), who report a minimum abstinence period of 24, 72 and 72 h, respectively. The mean age for ecstasy user group across studies was 23.39 years, with an average of 47.72% females. Mean lifetime dose across studies was 346.03 tablets. The mean age of the control group was 23.11 years, with an average of 54.67% females.

#### Studies

Studies comparing ecstasy users and controls in performance on behavioural tasks that are function specific were eligible for inclusion. The EFs included in this analysis were: updating; inhibitory control; switching; and access. Tasks eligible for inclusion can be seen in Table 1. There were no date limitations on publication.

**Table 1.** Tasks included for assessment of each executive function

Executive function	Task	Outcome measure
Inhibitory control	Stroop	Stroop interference RT
	RLG	Composite task score (reverse scored)
	Go No-Go	No-Go errors
		Or No-Go correct responses (reverse scored)
	Eriksen flanker task	Interference cost
	Stop signal	Stop signal RT
Switching	Stroop switch	Switch RT
	ToL	Total movements/solution time/proportion of perfect solutions
		Or solution time
	3D ID-ED	Simple reversal (switch cost)
	WCST	Perseverative errors
	Trail Making Test B	Time
	Stockings of Cambridge	
	Number–letter task	Switch cost
	Plus-minus task	Switch cost
	Dots-triangles task	Switch cost
	Local-global task	Switch cost
	Rule shift cards test	Task score
Updating	Keep track	Words
	Computation span	Task score
	Consonant/letter updating	Composite score
	Spatial updating	Composite score
	Digit span backwards	Task score
	2-Back letters	Correct responses
	2-Back figures	Correct responses
	Spatial span backwards	Task score
	Subtracting serial sevens	Errors
	Mental counters	Correct responses
Access	COWA/FAS/word fluency	Total words
	CWFT – C letter words	Total words
	CWFT – standardized score	Composite score
	Semantic retrieval task	Low association errors

RT, Reaction time; RLG, random letter generation; ToL, Tower of London; 3D ID-ED, three-dimensional Intra-dimensional/extra-dimensional task; WCST, Wisconsin Card Sorting Test; COWA, Controlled Oral Word Association; CWFT, Chicago Word Fluency Test.

#### Outcome measures

As each EF can be assessed using several tasks, there are a number of outcome measures. The outcome measure from each task that most clearly taps its putative EF was selected for inclusion in the analysis. As such, each task contributes one outcome measure to the analysis only. Tasks included as well as the outcome measure selected can be seen in Table 1.

### Data search and extraction

#### Information sources and search strategy

The formal search strategy involved searching three electronic databases during July 2015: PsycINFO, Scopus and Web of Science. Systematic searches used the key terms ‘Ecstasy’ OR ‘MDMA’ AND ‘executive function’. Supplementary searches were also conducted using the terms ‘Ecstasy’ and ‘MDMA’ combined with the name of each task in Table 1. Manual searches of reference sections of initially identified studies were conducted to supplement the formal electronic search; furthermore, articles that were not identified in the initial searches that the authors knew to be eligible for inclusion were assessed for inclusion. These additional searches yielded a further five studies eligible for inclusion.

#### Article selection and extraction of data

Initial searches were carried out by one author (C.A.R.). However, supplementary searches and manual searches were carried out by two authors (C.A.R. and C.M.). Both authors were responsible for the assessment of articles for inclusion, and decisions over article inclusion were made through discussion. One author (C.A.R.) extracted the relevant data and a second author (C.M.) cross-checked this. Several studies met inclusion criteria, but did not report sufficient information in the papers to compute the effect size; in each case data were requested from the corresponding author of the study. Data requests were not met for five articles: Semple *et al.* (1999); Thomasius *et al.* (2003); McCann *et al.* (2007); McCann *et al.* (2008); and Fagundo *et al.* (2010).

#### Additional handling of data

Composite performance scores for letter updating, spatial updating and random letter generation were calculated from the available data, if the composite score itself was not reported in the paper. On occasions where reported values of behavioural performance were split by gender, a weighted mean by number in each sample was calculated. A weighted s.d. was also calculated by multiplying squared s.d.s by number in each group, adding these together, then dividing by total *n*. The square root of this total was then used as the s.d. in analysis. Data for the FAS task were provided by Morgan *et al*. (2002), with means and s.d.s given for each letter. Therefore means for performance on each letter were added up to give a total score and the s.d.s were summed and divided by 3.

There were a number of cases where an article had used more than one task to assess an EF (Fox *et al*. 2001; Gouzoulis-Mayfrank *et al.* 2003; Montgomery *et al.* 2005*a*, 2007; Wareing *et al*. 2005; Lamers *et al*. 2006; Montgomery & Fisk, 2008; Fisk & Montgomery, 2009; Halpern *et al*. 2011). In these cases, means and s.d.s were entered for each task; however, the number of participants in each group was divided by the number of tasks included for that function from that paper.

In de Sola *et al.* (2008*a*, *b*), between-group comparisons were given 1 year apart. For the meta-analysis, we used baseline measurements of lifetime drug use and task performance. In cases where ecstasy user groups were broken down into further subgroups, e.g. ‘heavy and ‘light’ users (as per Fisk & Montgomery, 2009), data from the heavy user group were included in the analysis. In Fox *et al.* (2001) the user groups were split into problem/non-problem users and low/medium/high-intensity users. The group of high-intensity users was included in the current analysis. Although the ‘heavy’ and ‘high-intensity’ user group criteria were arbitrarily decided in the original papers, it seemed pertinent to include the user groups with the heaviest background ecstasy use in the current analysis, as these would be the most likely to show ecstasy-related cognitive impairment.

#### Data items extracted for individual studies

From each of the published papers, the following information was extracted for each group: number of participants; gender split; age; estimated lifetime dose of ecstasy; time since last use; task used (Table 2); outcome measure (Table 1); and means and s.d.s for each outcome variable. In cases where mean ecstasy abstinence duration was not reported, the minimum abstinence period required for the study was recorded. If not reported in the paper, estimates of mean lifetime dose of ecstasy were calculated from the available data. Reported ecstasy user groups could generally be defined by two categories: current users and former users. There were several categories of control groups, including: cannabis-only users; polydrug control groups (who had been recruited due to them having some degree of matching for other substances); non-users (this was a general catch-all name given to controls who were ecstasy naive but did have some other drug use); and drug-naive controls (no illicit substance use, but allowed for use of alcohol and nicotine).

**Table 2.** Summary of studies included in meta-analysis on executive function in current ecstasy users and drug-using controls^*a*^

Authors and study	Participants and design	Task(s) used	Result
Bedi & Redman (2008)	45 Ecstasy polydrug users (47% F, mean age 22.8 ± 3.0 years, MLD = 170.6 ± 362.8 tablets, MTSLU = 79.2 ± 108.5 days) 48 Cannabis polydrug users (46% F, mean age 21.7 ± 3.5 years)	COWA FAS	No between-group differences in original analysis
Croft *et al.* (2001)	11 MDMA and cannabis users (55% F, mean age 27.5 ± 4.7 years, MLD = 41.9 ± 49.3 occasions, no ecstasy abstinence data given) 18 Cannabis users (22% F, mean age 26.6 ± 8.1 years)	COWA FAS Stroop Digit span backwards	No differences in performance between MDMA users and cannabis users
Dafters (2006*a*)	33 Ecstasy and cannabis users (36% F, mean age 23.09 ± 2.34 years, MLD = 499.1 ± 671.56 tablets, minimum abstinence = 5 days) 18 Non-users (44% F, mean age 22.67 years)	Stroop Stroop switch Keep Track	Ecstasy users significantly impaired on task-switching Stroop, but not in Stroop interference or Keep Track task
Dafters (2006*b*)	18 Ecstasy and cannabis users (33% F, mean age 23.24 ± 2.33 years, MLD = 522.33 ± 936.71 tablets, minimum abstinence = 5 days) 18 Non-users (44% F, mean age 22.67 ± 2.56 years)	Stroop	No significant between-group differences
de Sola *et al.* (2008*a*)	37 Ecstasy polydrug users (49% F, mean age 23.6 ± 3.5 years, MLD = 206 ± 228.3 tablets, minimum abstinence = 72 h) 23 Cannabis users (65% F, mean age 22.0 ± 1.9 years)	ToL	No significant between-group differences at baseline
de Sola *et al.* (2008*b*)	14 Ecstasy polydrug users (57% F, mean age 25.2 ± 3.3 years, MLD = 207.4 ± 151.0 tablets, no abstinence data given) 13 Cannabis users (61% F, mean age 25.1 ± 2.9 years)	ToL	No significant between-group differences at baseline
Fisk & Montgomery (2009)	14 Heavy ecstasy users (36% F, mean age 22.86 years, MLD = 1000.21 ± 786.41 tablets, MTSLU = 22 weeks) 28 Non-users (75% F, mean age 20.71 years)	RLG Computation span Consonant updating Spatial updating	Heavy users not impaired at RLG. All updating measures show ecstasy-related deficits, and these were significant in two out of three measures
Fisk *et al.* (2004)	44 Ecstasy users (mean age 21.52 ± 1.66 years, MLD = 343.38 ± 376.94 tablets, MTSLU = 10.90 ± 27.86 weeks) 59 Non-users (mean age 21.37 ± 1.84 years)	RLG Computation span	No group differences on RLG performance. Ecstasy users significantly impaired on computation span
Fox *et al.* (2001)	11 High-intensity ecstasy users (45% F, mean age 28.0 ± 5.3 years, MTSLU = 2.8 ± 5.9 months) 20 Polydrug controls (70% F, mean age 23.3 ± 6.5 years)	WCST ToL	No between-group differences in WCST perseverative errors or ToL solution time
Fox *et al.* (2002)	20 Ecstasy polydrug users (50% F, mean age 27.3 ± 6.7 years, MLD = 172.0 ± 227.36 tablets, MTSLU = 51.9 ± 25.9 months) 20 Polydrug controls (60% F, mean age 27.5 ± 7.6 years)	3D ID-ED	No between-group differences
Gouzoulis-Mayfrank *et al.* (2000)	28 Ecstasy users (43% F, mean age 23.25 years, MLD = 93.4 ± 119.9 tablets, MTSLU = 41 ± 71.1 days) 28 Polydrug controls (46% F, mean age 22.9 years)	Stroop Digit span backwards Phonological word fluency	Ecstasy users performed worse than non-users in digit span backwards. No performance differences observed in Stroop interference or word fluency
Gouzoulis-Mayfrank *et al.* (2003)	30 Heavy ecstasy users (30% F, mean age 25.1 ± 4.65 years, MLD = 503.2 ± 555.5 tablets, MTSLU = 194.8 ± 351.8 days) 30 Non-users (30% F, mean age 25.37 ± 2.72 years)	Go No-Go Digit span backwards 2-Back letters 2-Back figures	No differences between ecstasy users and controls in central executive function
Halpern *et al.* (2004)	23 Ecstasy users with minimal exposure to other drugs (65% F, mean age 20 years, MLD = 60 episodes) 16 Controls equally involved in rave culture (44% F, mean age 22 years)	COWA FAS Stroop WCST Digit span backwards	No between-group differences in FAS, WCST, Stroop or digit span backwards. However, ecstasy-related impairment on digit span backwards when adjusted for age and sex
Halpern *et al.* (2011)	52 Ecstasy users (46% F, mean age 22 years, MLD = 43.5 episodes, MTSLU = 121 days) 59 Non-users (36% F, mean age 24 years)	Spatial span backwards Digit span backwards Stroop WCST TMT-B	No significant between-group differences on any of the executive measures
Hanson & Luciana (2004)	26 Ecstasy users (46% F, mean age 21.3 ± 3.6 years, MLD = 123.31 occasions, MTSLU = 10.9 ± 10.5 weeks) 26 Non-users (46% F, mean age 20.7 ± 3.4 years)	COWA FAS Digit span backwards	No between-group differences in COWA total words, or digit span backwards performance
Heffernan *et al.* (2001)	30 Regular ecstasy users (43% F, mean age 23.9 ± 4.47 years, minimum TSLU = 24 h) 37 Ecstasy-free controls (73% F, mean age 25.5 ± 8.76 years)	Word fluency, C letter words	Ecstasy users performed significantly worse than controls in verbal fluency measure
Hoshi *et al.* (2007)	25 Ecstasy users (mean age 28.64 ± 4.59 years, MLD = 1111.68 tablets, MTSLU = 14.2 days) 29 Polydrug users (mean age 31.93 ± 8.41 years)	Subtracting serial sevens Verbal fluency TMT-B Go/No-Go	No significant group differences were found in Serial Sevens, verbal fluency, the TMT
Lamers *et al.* (2006)	11 MDMA/THC users (mean age 22.9 ± 2.4 years, MTSLU = 228.1 ± 140.3 days) 15 Cannabis users (mean age 24.3 ± 5.3 years)	TMT-B WCST	No between-group effects on TMT-B or WCST
McCardle *et al.* (2004)	17 Ecstasy users (24% F, mean age 21.06 ± 1.56 years, MTSLU = 130 days) 15 Controls (13% F, mean age 21.91 ± 1.62 years)	Digit span backwards TMT-B	No between-group effects observed in digit span backwards or TMT-B
Montgomery & Fisk (2008)	73 Ecstasy polydrug (47% F, mean age 21.77 ± 2.11 years, MLD = 309.86 ± 486.25 tablets, MTSLU = 32.15 ± 62.82 weeks) 73 Non-ecstasy users (73% F, mean age 20.73 ± 1.73 years)	Letter updating Spatial updating	Ecstasy users impaired in four out of six subsample analyses
Montgomery *et al.* (2005*a*)	Study 1: 27 ecstasy users (48% F, mean age 21.70 ± 1.66 years, MLD = 345.96 ± 365.76 tablets, MTSLU = 4.97 ± 7.27 weeks) 34 Non-users (71% F, mean age 21.59 ± 1.88 years) Study 2: 51 ecstasy users (47% F, mean age 21.96 ± 2.11 years, MLD = 373.87 ± 542.91 tablets, MTSLU = 22.15 weeks) 42 Non-users (79% F, mean age 20.83 ± 1.45 years)	CWFT C letter words Computation span Letter updating Number–letter task Plus–minus task RLG	Ecstasy users performed worse on both updating tasks and access to long-term memory tasks Ecstasy users performed significantly better on the inhibition task. No group differences were observed in switching
Montgomery *et al.* (2005*b*)	22 MDMA users (50% F, mean age 21.36 ± 1.67 years, MLD = 303.3 ± 374.04 tablets, MTSLU = 4.61 ± 6.82 weeks) 26 Non-MDMA users (62% F, mean age 21.31 ± 1.69 years)	RLG – task score (inhibition) Computation span – task score (updating)	Ecstasy users performed significantly worse than non-users in the computation span task. There were no group differences in RLG performance
Montgomery *et al.* (2007)	104 Ecstasy users (mean age 21.68 ± 1.96 years, MLD = 349.97 ± 464.41 tablets, MTSLU = 19.35 ± 43.46 weeks) 103 Non-users (mean age 21.11 ± 1.66 years)	CWFT Computation span Letter updating	Ecstasy users performed worse than controls on all measures
Morgan (1998)	Study 1: 16 ecstasy users (50% F, mean age 20.94 ± 1.88 years, MLD = 35.5 ± 17.5 tablets, MTSLU = 20.4 ± 33.6 days) 12 Polydrug controls (mean age 20.25 ± 1.48 years) Study 2: 25 ecstasy users (52% F, mean age 22.28 ± 2.48 years, MLD = 49.6 ± 33.2 occasions, MTSLU = 65.1 ± 85.7 days) 20 Polydrug controls (mean age 23 ± 4.71 years)	ToL	No between-group differences of ToL performance in either study
Morgan *et al.* (2002)	18 Ecstasy users (50% F, mean age 23.4 ± 3.2 years, MLD = 303 ± 267.5 tablets, MTSLU = 4.05 ± 3.2 weeks) 16 Polydrug users (50% F, mean age 22.1 ± 3.3 years)	TMT-B COWA FAS Stroop Subtracting serial sevens	Ecstasy users worse on Subtracting serial sevens than all groups. However, no between-group differences observed in verbal fluency, Stroop interference reaction time, or TMT-B completion time
Murphy *et al.* (2011)	15 Ecstasy and cannabis users (73% F, mean age 24.5 ± 3.4 years, MLD = 364.8 ± 665.1 tablets, MTSLU = 365 days) 13 Cannabis users (54% F, mean age 21.9 ± 4.6 years)	RLG	Ecstasy users had significantly higher redundancy on RLG than drug-naive controls but not cannabis controls
Nulsen *et al.* (2011)	11 Ecstasy users (64% F, mean age 22.9 ± 2.6 years, MLD = 32.5 ± 27.2 occasions) 13 Polydrug controls (70% F, mean age 23.2 ± 3.3 years)	Digit span backwards	No significant between-group differences in digit span backwards performance
Reay *et al.* (2006)	15 Ecstasy polydrug users (40% F, mean age 25 ± 5.8 years, MLD = 593.4 tablets) 15 Polydrug controls (53% F, mean age 21.3 ± 538 years)	Digit span backwards Brixton spatial anticipation task Inhibition of return	Ecstasy users performed significantly worse on digit span backwards and the Brixton spatial anticipation task. No between-group differences observed in inhibition of return
Reneman *et al.* (2006)	23 Heavy ecstasy (48% F, mean age 26.05 ± 5.05 years, MLD = 516.35 ± 452.1 tablets, MTSLU = 2.29 ± 2.39 months) 15 Polydrug controls (53% F, mean age 26.3 ± 4.1 years)	COWA FAS Stroop WCST TMT-B	No between-group differences overall on executive functioning
Roberts *et al.* (2013*a*)	20 Ecstasy polydrug users (50% F, mean age 23.95 ± 2.50 years, MLD = 177.65 ± 301.73 tablets, minimum abstinence = 7 days) 20 Polydrug controls (55% F, mean age 22.58 ± 3.45 years)	Go/No-Go	No between-group differences in No-Go errors
Roberts *et al.* (2013*b*)	20 Ecstasy polydrug users (50% F, mean age 23.95 ± 2.50 years, MLD = 177.65 ± 301.73 tablets, minimum abstinence = 7 days) 20 Polydrug controls (55% F, mean age 22.58 ± 3.45 years)	Semantic retrieval task	No behavioural between-group differences
Roberts *et al.* (2013*c*)	20 Ecstasy polydrug users (50% F, mean age 23.95 ± 2.50 years, MLD = 177.65 ± 301.73 tablets, minimum abstinence = 7 days) 20 Polydrug controls (55% F, mean age 22.58 ± 3.45 years)	Number–letter task	No behavioural between-group differences
Rodgers (2000)	15 Ecstasy users (53% F, mean age 31 years 5 months, MLD = 20 occasions, minimum abstinence = 2 months) 15 Cannabis users (53% F, mean age 30 years 3 months)	Digit span	No performance difference in digit span
von Geusau *et al.* (2004)	26 Ecstasy users (35% F, mean age 21.55 ± 1.3 years, minimum abstinence = 2 weeks) 33 Non-users (64% F, mean age 21.7 ± 2.1 years)	WCST ToL Stop signal task Mental counters	Male MDMA users performed worse on tasks that tap cognitive flexibility. No differences were observed on other cognitive tasks. Female users showed no impairments
Wareing *et al.* (2004)	42 Ecstasy users (48% F, mean age 21.69 ± 2.57 years, MLD = 552.99 ± 681.41 tablets, MTSLU = 3 ± 3.66 weeks) 31 Non-users (61% F, mean age 23.39 ± 6.47 years)	Computation span	MDMA users performed significantly worse than controls on computation span task
Wareing *et al.* (2005)	36 Ecstasy users (mean age 21.81 years, MLD = 591.33 ± 718.44 tablets, MTSLU = 3.30 ± 3.87 weeks) 31 Non-users (mean age 23.39 ± 6.47 years)	Spatial working memory span Computation span	Ecstasy users (users and former users) show impaired spatial working memory compared with controls
Wareing *et al.* (2007)	29 Ecstasy users (mean age 21.72 ± 2.00 years, MLD = 536 ± 515.73 tablets, MTSLU = 1.86 ± 1.50 weeks) 46 Non-users (mean age 22.85 ± 5.50 years)	Computation span	Both ecstasy user groups performed significantly worse than non-users on the computation span measure
Yip & Lee (2005)	100 Ecstasy users (mean age 28.48 ± 5.71 years, MLD = 35.81 ± 13.21 tablets, MTSLU = 2.23 ± 0.51 months) 100 Non-users (mean age 28.82 ± 5.78 years)	Stroop Digit span backwards	No between-group differences on backwards digit span. However, ecstasy users performed significantly worse at the Stroop task
Zakzanis & Young (2001)	30 Ecstasy users (67% F, mean age 22.96 years, MLD = 37.76 occasions, MTSLU = 19.96 weeks) 24 Non-users (67% F, mean age 19.54 years)	Rule shift cards test	No significant difference between groups in rule shift cards test performance

F, Female; MLD, mean lifetime dose; MTSLU, mean time since last use; COWA, Controlled Oral Word Association; MDMA, 3,4-methylenedioxymethamphetamine; ToL, Tower of London task; RLG, random letter generation; WCST, Wisconsin Card Sorting Test; 3D ID-ED, three-dimensional Intra-dimensional/extra-dimensional task; TMT-B, Trail Making Test B; THC, tetrahydrocannabinol; CWFT, Chicago Word Fluency Test.

^a^ For information on previous exposure to other drugs and other groups not included in the meta-analysis, see online Supplementary Table S1.

### Statistical and subgroup analysis

Standardized mean difference (SMD) and standard error (s.e.) of the SMD between experimental conditions were calculated for each executive task outcome separately in each study. SMDs were employed due to variation in outcome measures in the behavioural tasks included in the analysis. SMD estimates differences between two experimental conditions on an outcome variable (SMD = mean1 – mean2/pooled s.d.). This allowed for a subgroup analysis to be conducted by EF (inhibitory control, updating, access and switching). The meta-analysis used generic inverse variance methods to synthesize individual SMDs, in the software package RevMan 5.2 (The Nordic Cochrane Centre, Copenhagen). The magnitude of SMDs can be interpreted thus: 0.2 = small, 0.5 = moderate, and 0.8 = a large effect (Higgins & Green, 2011).

#### Analytic strategy

The meta-analysis was conducted by separating effect sizes from tasks employed in each study into distinct EFs. The main effect and formal subgroup analysis was examined, whereby each EF was considered a subgroup.

Outcome measures of the various tasks that were included in this meta-analysis had to be reviewed by the authors so that the direction of differences in task performance were consistent for interpretation of ecstasy-related impairment. For example, if ecstasy users produced fewer words on the verbal fluency tasks relative to controls, this would be indicative of ecstasy-related impairment in verbal fluency and would result in a negative SMD in the meta-analysis. However, a greater amount of perseveration errors on the Wisconsin Card Sorting Test would be indicative of impairment yet would yield a positive SMD, should ecstasy users produce more errors here. As such, outcome measures were negatively coded where appropriate.

The main analysis was conducted on the 39 studies that assessed one or more EF in a current ecstasy user group *versus* a control group that had some use of recreational drugs. Studies that employed a drug-naive control group and no-drug user control group were not included in the analysis, with the exception of three studies (Halpern *et al.* 2004, 2011; Yip & Lee, 2005). These studies were included, with a drug-naive control group, as their current ecstasy user groups had minimal exposure to other drugs. The remaining studies featured a drug-using control group; as such, all between-group comparisons in this meta-analysis have at least some degree of matching for other drug use. Random-effects models were employed due to high heterogeneity in the data across studies.

## Results

### Study selection (Fig. 1)

**Fig. 1.**
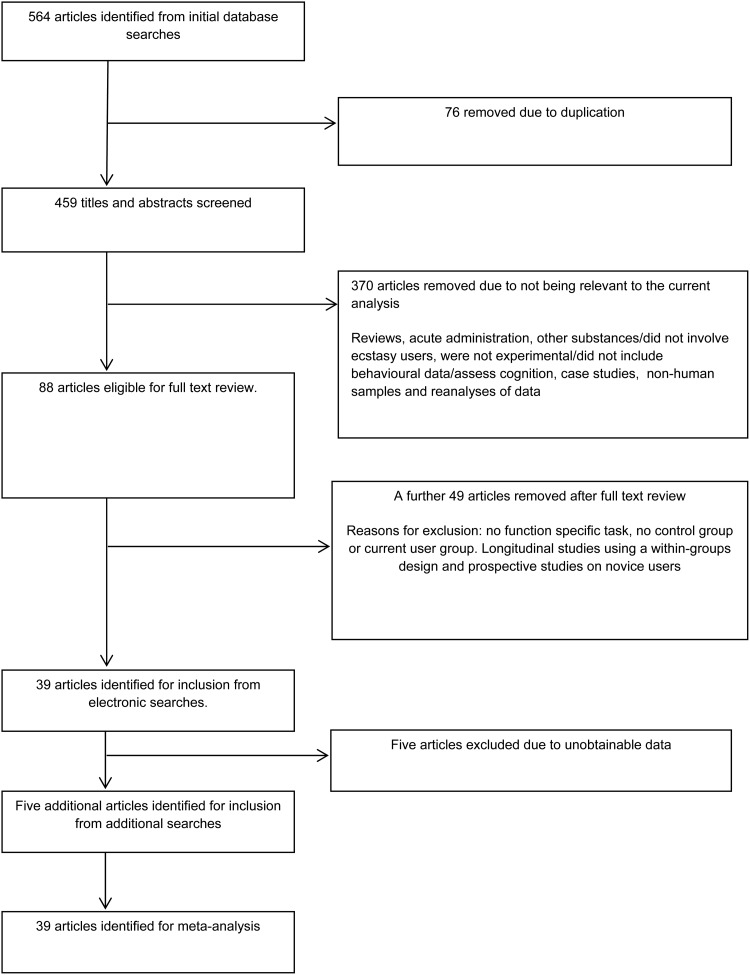
Meta-analysis search results and flow chart.

Initial literature searches yielded 99 papers using Web of Science, 79 using Scopus and 386 papers from PsycINFO. After removing 76 duplicated papers, 459 articles remained. A brief review of the remaining article titles and abstracts led to exclusion of 370 irrelevant articles. Excluded papers at this stage included: review articles (23); acute administration studies (26); studies that were conducted using other substances/did not involve ecstasy users (75); studies that were not experimental/did not include behavioural data/assess cognition (232); case studies (8); studies conducted in non-human samples (4); a study not written in English (1); and reanalyses of data (2). This left a total of 88 articles for full review. Further studies were excluded at this stage if they did not employ a function-specific task identified in Table 1 (35), did not employ a control group or current user group, or did not conduct between-group analysis (10). Longitudinal studies using a within-groups design and prospective studies on novice users were also excluded at this stage (4). Following these data exclusion procedures, 39 studies remained. A further five studies eligible for inclusion were identified from supplementary searches. Of the 44 studies that met all the inclusion criteria, data were not available for five; as such, the final meta-analyses were conducted on data from 39 articles (Fig. 1).

### Overview

#### Participant characteristics

Individual study information, including sample sizes and participant characteristics, is given in Table 2.

### Meta-analysis on EF in ecstasy polydrug users

Data from 39 published studies, contributing 89 effect sizes, were included in analysis, including data from a total of 1221 current ecstasy users and 1242 controls. For descriptive information from each study, see Table 2.

#### Meta-analyses (Fig. 2)

The test for overall effects was significant [SMD = −0.18, 95% confidence interval (CI) −0.26 to −0.11, *Z* = 5.05, *p* < 0.001, *I*^2^ = 82%], suggesting an overall executive performance deficit in ecstasy users relative to controls, albeit a small effect. However, there was also a significant subgroup effect (χ^2^ = 22.06, degrees of freedom = 3, *p* *<* 0.001, *I*^2^ = 86.4%) demonstrating differential effects across EFs. Individual analyses are reported below.

#### Access

A total of 13 studies, contributing 13 effect sizes, assessed access to long-term/semantic memory, with a total of 483 ecstasy users and 491 controls. A significant difference was observed between these two comparison groups (SMD = −0.33, 95% CI −0.46 to −0.19, *Z* = 4.72, *p* *<* 0.001, *I*^2^ = 74%), demonstrating that ecstasy users perform poorly compared with controls in this EF.

#### Inhibition

A total of 20 studies, contributing 20 effect sizes, investigated performance difference in inhibitory control providing a comparison between 606 ecstasy users and 632 controls. No between-group difference was observed in performance of this EF (SMD = 0.04, 95% CI −0.07 to 0.15, *Z* = 0.77, *p* *>* 0.05).

#### Switching

Switching was assessed in a total of 488 ecstasy users and 459 controls, in a total of 18 papers, contributing 23 effect sizes. There were significant between-group differences in this function (SMD = −0.19, 95% CI −0.36 to −0.02, *Z* = 2.16, *p* *<* 0.05, *I*^2^ = 85%), demonstrating that ecstasy use leads to impairment in mental set switching.

#### Updating

A total of 872 ecstasy users and 904 controls were compared for updating performance from a total of 24 articles, contributing 33 effect sizes. Again, there was a significant between-group difference in performance of updating tasks (SMD = −0.26, 95% CI −0.37 to −0.15, *Z* = 4.49, *p* *<* 0.001, *I*^2^ = 82%). This demonstrates that there is an ecstasy-related impairment with regards to updating performance.

**Fig. 2.**
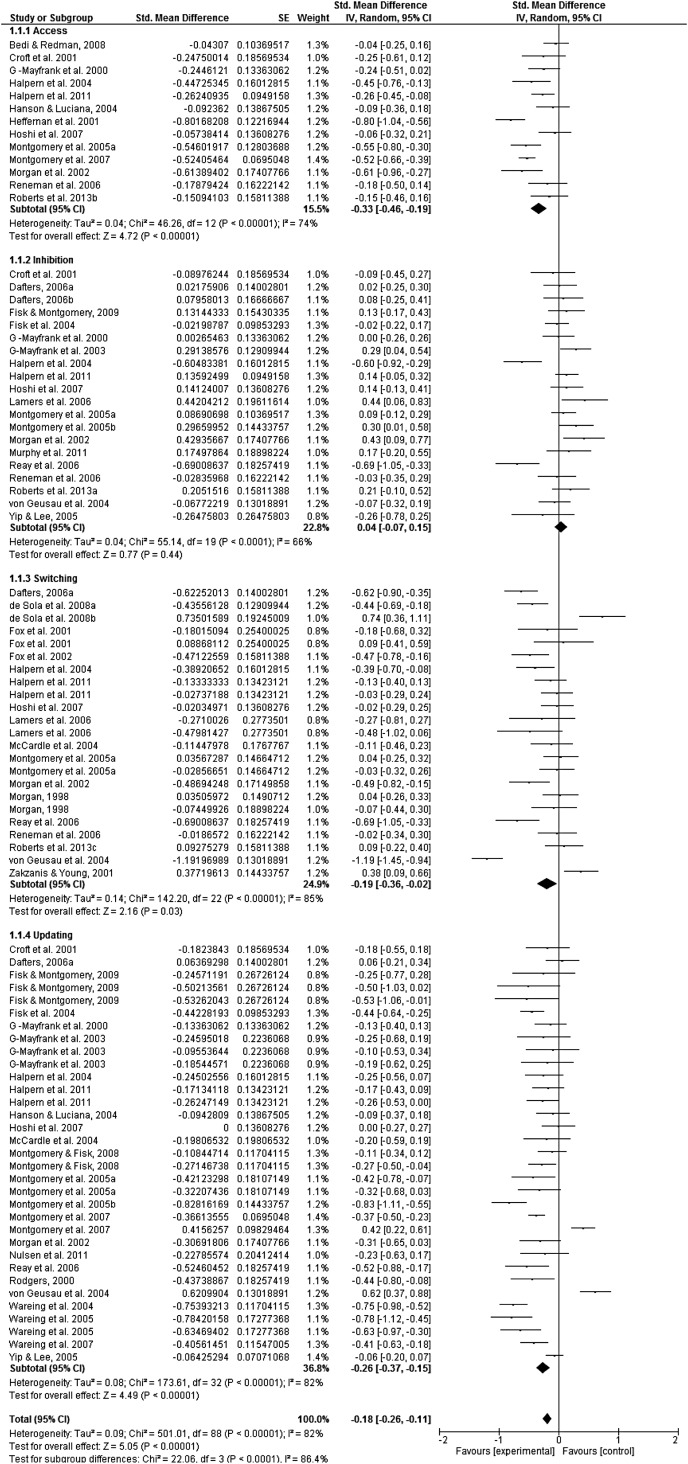
Forest plot of studies assessing executive function in ecstasy users and drug-using controls. *I*^2^ is an indicator of heterogeneity between comparisons. Inverse variance (IV) meta-analysis using standardized (Std.) mean differences. SE, Standard error; CI, confidence interval; df, degrees of freedom.

#### Meta-regression

We conducted a method of moments (random-effect model) meta-regression across the 64 comparisons included in the main meta-analysis, with the available data for estimates of lifetime dose of ecstasy. This was conducted to observe whether there was a relationship between lifetime dose of ecstasy and SMD in executive performance. The overall meta-regression was non-significant (regression coefficient −0.0001, 95% CI −0.0004 to 0.0002, *Z* = −0.74, *p* *>* 0.05), suggesting that lifetime dose did not predict performance differences. Furthermore, individual meta-regressions performed separately for each specific EF were all non-significant (*p* > 0.05 in each case).

### Evidence of publication bias

Examination of a funnel plot revealed asymmetry; therefore an Egger's test of publication bias was conducted (Egger *et al.* 1997) on the 89 effect sizes included in this meta-analysis. Egger's test was significant (*t*_88_ = −1.96, *p* = 0.05), suggesting evidence of publication bias. However, these results should be interpreted with caution due to the high heterogeneity between studies (Sterne *et al.* 2011).

## Discussion

The results from this meta-analysis demonstrate EF deficits in current ecstasy users. However, the size of this overall effect was small. Subgroup analyses showed that effect sizes varied by the specific component of executive functioning. Individual analyses by function showed ecstasy-related deficits in the EFs access, switching and updating, though there was no inhibition performance deficit.

Meta-regression using estimated lifetime dose of ecstasy to predict effect size of between-group differences was non-significant. This suggests that lifetime dose is not the greatest predictor in magnitude of EF deficit. However there were nine studies (providing 25 comparisons) that did not give lifetime estimates of use and so were not included in the analysis, which may have potentially given a different outcome. Nevertheless, there was high variability in effects and although estimates of lifetime use were not possible for all studies, there were 64 comparisons from 30 studies which did include estimated lifetime dose, which is far greater than the minimum of 10 required for adequate power in a meta-regression (Borenstein *et al.* 2009). Despite adequate power to detect an effect, it could be that the analysis is conceptually flawed, given that it is conducted on SMDs in performance between ecstasy users and controls rather than estimated lifetime dose and task performance (Murphy *et al.* 2012). Alternatively, it could be that there are other ecstasy-using behaviours that have a stronger impact on behavioural measures, for example recency of use, frequency of use and higher nightly doses. Recency of use has been identified as a predictor of haemodynamic response to a cognitive task in ecstasy users (Roberts & Montgomery, 2015*b*). Furthermore, higher nightly doses may make an impact on cognition more than cumulative intake; indeed a single high dose of MDMA is enough to cause neurotoxicity in laboratory animals (Molliver *et al.* 1990). Unfortunately, there is substantial variance in the reporting of drug use histories in the literature, limiting interpretation. Perhaps some unity on background drug use reporting would vastly improve research and our understanding of harmful behaviours. We propose that a unified reporting criterion should be applied to future research. There are also a number of variables that may contribute to the impact of cumulative dose (Murphy *et al.* 2012) including earlier onset of use, use of other drugs, and increased bioenergetic stress (Parrott, 2009).

Neuronal regions implicated in working memory and EF include the DLPFC and the hippocampus (depending on the nature of the task). These structures have dense innervation of serotonergic (5-HT) neurons (Pazos *et al.* 1987; Curtis & D'Esposito, 2003). Therefore ecstasy-related degradation to the serotonin system, through neurotoxicity or down-regulation following chronic recent use, is understood to be a potential cause of cognitive impairment in the functions supported by these areas. If ecstasy is a serotonin-specific neurotoxin in humans as it is in animals (Green *et al.* 2003), one would expect functional alterations following repeated use. Several molecular imaging studies in human ecstasy users suggest a reduction in pre-synaptic serotonin transporter (SERT) availability in areas including the frontal cortex (McCann *et al.* 1998; Kish *et al.* 2010) and the DLPFC (McCann *et al.* 2005). Increases in post-synaptic 5-HT_2A_ receptors have also been observed in ecstasy users relative to controls in the DLPFC (Urban *et al.* 2012). Decreased pre-synaptic SERT and increased post-synaptic 5-HT_2A_ receptor availability are consistent with serotonin axon damage. Moreover, functional neuroimaging studies have observed ecstasy-related adjustments to cerebral blood flow in frontal areas, with functional near-infrared spectroscopy (Roberts & Montgomery, 2015*a*) and fMRI (Moeller *et al.* 2004; Jager *et al.* 2008; Roberts & Garavan, 2010). It is noteworthy that all of the functional imaging studies mentioned observe increased neuronal activity to achieve similar behavioural performance to controls. This suggests that molecular and functional neuroimaging detect changes in serotonin signalling which cause future deficits in EF. The current results support this by demonstrating behavioural correlates for the supposed neuronal degradation.

Ecstasy-related impairments in switching were unexpected, given that previous reviews in this area have concluded that this function is relatively stable (Murphy *et al.* 2009). However, some studies have observed significant switching differences between ecstasy users and controls (Halpern *et al.* 2004; Dafters, 2006*a*) and neuroimaging studies have suggested atypical processing during switching (Roberts *et al*. 2013*c*). This highlights the necessity for larger samples to elucidate this performance deficit. However, this difference was the weakest of the three significant differences and had a small effect size; thus it should be treated with caution. The reduced performance in updating and access in ecstasy users relative to controls is more consistent with previous reports (Montgomery *et al.* 2005*a*; Murphy *et al.* 2009). Nevertheless, there have been previous reports of null findings in these functions. The ability to update one's memory is reflective of the concept of working memory as a whole, and Miyake and co-workers (Friedman *et al.* 2006; Miyake & Friedman, 2012) maintain that updating is the key overarching EF which is important for daily function.

Although not unexpected, it is interesting to consider why there were no apparent group differences in inhibitory control. One explanation could be that ecstasy users are high-functioning impulsives and this increased impulsivity serves to mask performance deficits on the tasks employed here (Fritzsche *et al.* 2011). Alternatively, perhaps inhibitory control impairment is associated with other psychostimulants that are primarily dopaminergic in nature, e.g. cocaine (Fillmore & Rush, 2002) and methamphetamine (Monterosso *et al.* 2005). Interestingly, in recent models of the unity and diversity of EFs, Miyake & Friedman (2012) confer that inhibitory control is not necessarily a unique EF. Instead, inhibitory control is subsumed by common EF ability. With this in mind, it could be suggested that ecstasy users are therefore impaired at each level of EF.

There are a number of limitations of the current analysis. Concomitant use of other drugs is often posited to contribute to the cognitive deficits displayed by ecstasy users. To try and incorporate this into the meta-analysis, comparisons were made between ecstasy users and controls that have at least some experience with drugs other than ecstasy. Nevertheless, it should be noted that in many of the studies in the analysis, the use of drugs other than ecstasy was, in fact, higher in the ecstasy user groups than the polydrug control groups (in terms of total lifetime dose, frequency of use and variety of drugs used). As such, we cannot rule out the possibility that alcohol and other drugs may also contribute to deficits in executive functioning. However, despite the increased polydrug use among ecstasy user groups, there are several instances of drug use indices predicting unique variance in EFs in regression analyses (for example, Schilt *et al.* 2008); this suggests that various chronic drug effects do show independence from one another. Increased cohesion in reporting of drug use variables would help to remove some of this uncertainty in future. Similarly, it cannot be ruled out that the direction of causality is interpreted incorrectly. It could be that individuals with EF deficits are more likely to have a stronger propensity for ecstasy use, though the authors think that this is unlikely. Future research should concentrate on longitudinal studies to obviate confusion over direction of causality. Furthermore, as the current analysis is conducted on current users and therefore cannot make any predictions about function recovery following abstention, longitudinal studies may also help to determine whether recovery is possible. The current results suggest that ecstasy users may struggle with higher-level executive functioning, and it has been suggested that such impairments would lead to difficulty in performing the majority of occupational tasks (Parrott, 2013*a*, *b*). Montgomery *et al.* (2010) observed ecstasy users to be impaired at a virtual reality office work task, with the suggestion that office work, as well as those occupations requiring greater executive resources, will be adversely affected by ecstasy use. Taken together, these findings suggest that prolonged ecstasy use can lead to everyday functioning problems; therefore an understanding of the processes underpinning such impairments may prove valuable to clinicians.

To conclude, the current meta-analysis demonstrated that EF performance in ecstasy users is significantly reduced overall compared with controls. The three functions that show significant impairment are updating, switching and access, whilst inhibitory control is unaffected by ecstasy use. This is the most comprehensive analysis of EF in ecstasy users to date and provides a behavioural correlate of potential serotonergic neurotoxicity.

## Supplementary material

For supplementary material accompanying this paper visit http://dx.doi.org/10.1017/S0033291716000258. click here to view supplementary material

## References

[ref1] Anderson T (2014). Molly deaths and the failed war on drugs. Contexts 13, 48–53.

[ref2] Baddeley A (2000). The episodic buffer: a new component of working memory? Trends in Cognitive Sciences 4, 417–423. 11058819 10.1016/s1364-6613(00)01538-2

[ref3] Bedi G, Redman J (2008). Ecstasy use and higher-level cognitive functions: weak effects of ecstasy after control for potential confounds. Psychological Medicine 38, 1319–1330. 18226286 10.1017/S0033291708002730

[ref4] Borenstein M, Hedges LV, Higgins JPT, Rothstein HR (2009). Introduction to Meta-Analysis. Wiley: Chichester.

[ref5] Burgess AP, Venables L, Jones H, Edwards R, Parrott AC (2011). Event related potential (ERP) evidence for selective impairment of verbal recollection in abstinent recreational methylenedioxymethamphetamine (“ecstasy”)/polydrug users. Psychopharmacology 216, 545–556. 21390504 10.1007/s00213-011-2249-9

[ref6] Chambers CD, Garavan H, Bellgrove MA (2009). Insights into the neural basis of response inhibition from cognitive and clinical neuroscience. Neuroscience and Biobehavioural Reviews 33, 631–646. 10.1016/j.neubiorev.2008.08.016 18835296

[ref7] Croft RJ, Mackay AJ, Mills ATD, Gruzelier JGH (2001). The relative contributions of ecstasy and cannabis to cognitive impairment. Psychopharmacology 153, 373–379. 11271410 10.1007/s002130000591

[ref8] Curran HV, Rees H, Hoare T, Hoshi R, Bond A (2004). Empathy and aggression: two faces of ecstasy? A study of interpretative cognitive bias and mood change in ecstasy users. Psychopharmacology 173, 425–433. 14735288 10.1007/s00213-003-1713-6

[ref9] Curtis CE, D'Esposito M (2003). Persistent activity in the prefrontal cortex during working memory. Trends in Cognitive Sciences 7, 415–423. 12963473 10.1016/s1364-6613(03)00197-9

[ref10] Dafters RI (2006 *a*). Chronic ecstasy (MDMA) use is associated with deficits in, task-switching but not inhibition or memory updating executive functions. Drug and Alcohol Dependence 83, 181–184. 16316723 10.1016/j.drugalcdep.2005.11.006

[ref11] Dafters RI (2006 *b*). Impulsivity, inhibition and negative priming in ecstasy users. Addictive Behaviors 31, 1436–1441. 16242244 10.1016/j.addbeh.2005.09.013

[ref12] Daumann J, Fischermann T, Heekeren K, Henke K, Thron A, Gouzoulis-Mayfrank E (2005). Memory-related hippocampal dysfunction in poly-drug ecstasy (3,4-methylenedioxymethamphetamine) users. Psychopharmacology 180, 607–611. 15372137 10.1007/s00213-004-2002-8

[ref13] de Sola Llopis S, Miguelez-Pan M, Peña-Cassanova J, Poudevida S, Farré M, Pacifici R, Böhm P, Abanades S, Verdejo-Garcia A, Zuccaro P, de la Torre R (2008 *a*). Cognitive performance in recreational ecstasy polydrug users: a two-year follow-up study. Journal of Psychopharmacology 22, 498–510. 18208910 10.1177/0269881107081545

[ref14] de Sola S, Tarancón T, Peña-Cassanova J, Espadaler JM, Langohr K, Poudevida S, Farré M, Verdejo-Garcia A, de la Torre R (2008 *b*). Auditory event-related potentials (P3) and cognitive performance in recreational ecstasy polydrug users: evidence from a 12-month longitudinal study. Psychopharmacology 200, 425–437. 18581098 10.1007/s00213-008-1217-5

[ref15] Egger M, Smith GD, Schneider M, Minder C (1997). Bias in meta-analysis detected by a simple, graphical test. British Medical Journal 315, 629–634. 9310563 10.1136/bmj.315.7109.629 PMC2127453

[ref16] Fagundo AB, Cuyás E, Verdejo-Garcia A, Khymenets O, Langohr K, Martín-Santos R, Farré M, de la Torre R (2010). The influence of 5-HTT and COMT genotypes on verbal fluency in ecstasy users. Journal of Psychopharmacology 24, 1381–1393. 20080926 10.1177/0269881109354926

[ref17] Fillmore MT, Rush CR (2002). Impaired inhibitory control of behaviour in chronic cocaine users. Drug and Alcohol Dependence 66, 265–273. 12062461 10.1016/s0376-8716(01)00206-x

[ref18] Fisk JE, Montgomery C (2009). Evidence for selective executive function deficits in ecstasy/polydrug users. Journal of Psychopharmacology 23, 40–50. 18515461 10.1177/0269881108089815

[ref19] Fisk JE, Montgomery C, Murphy P, Wareing M (2004). Evidence for executive deficits among users of MDMA (ecstasy). British Journal of Psychology 95, 457–466. 15527532 10.1348/0007126042369785

[ref20] Fisk JE, Sharp CA (2004). Age-related impairment in executive functioning: updating, inhibition, shifting and access. Journal of Clinical and Experimental Neuropsychology 26, 874–890. 15742539 10.1080/13803390490510680

[ref21] Fox HC, McLean A, Turner JJD, Parrott AC, Rogers R, Sahakian BJ (2002). Neuropsychological evidence of a relatively selective profile of temporal dysfunction in drug-free MDMA (‘ecstasy’) polydrug users. Psychopharmacology 162, 203–214. 12110998 10.1007/s00213-002-1071-9

[ref22] Fox HC, Parrott AC, Turner JJD (2001). Ecstasy use: cognitive deficits related to dosage rather than self-reported problematic use of the drug. Journal of Psychopharmacology 15, 273–281. 11769821 10.1177/026988110101500406

[ref23] Friedman NP, Miyake A, Corley RP, Young SE, DeFries JC, Hewitt JK (2006). Not all executive functions are related to intelligence. Psychological Science 17, 172–179. 16466426 10.1111/j.1467-9280.2006.01681.x

[ref24] Fritzsche AS, Stahl J, Gibbons H (2011). An ERP study of target competition: individual differences in functional impulsive behavior. International Journal of Psychophysiology 81, 12–21. 21510982 10.1016/j.ijpsycho.2011.03.014

[ref25] Goldman-Rakic PS (1996). The prefrontal landscape: implications of functional architecture for understanding human mentation and the central executive. Philosophical Transactions of the Royal Society of London. Series B, Biological Sciences 351, 1445–1453. 8941956 10.1098/rstb.1996.0129

[ref26] Gouzoulis-Mayfrank E, Daumann J, Tuchtenhagen F, Pelz S, Becker S, Kunert HJ, Fimm B, Sass H (2000). Impaired cognitive performance in drug free users of recreational ecstasy (MDMA). Journal of Neurology, Neurosurgery, and Psychiatry 68, 719–725. 10.1136/jnnp.68.6.719 PMC1736948 10811694

[ref27] Gouzoulis-Mayfrank E, Thimm B, Rezk M, Hensen G, Daumann J (2003). Memory impairment suggests hippocampal dysfunction in abstinent ecstasy users. Progress in Neuro-Psychopharmacology and Biological Psychiatry 27, 819–827. 12921915 10.1016/S0278-5846(03)00114-3

[ref28] Green AR, Mechan AO, Elliot JM, O'Shea E, Colado MI (2003). The pharmacology and clinical pharmacology of 3,4-methylenedioxymethamphetamine (MDMA, “ecstasy”). Pharmacological Reviews 55, 463–508. 12869661 10.1124/pr.55.3.3

[ref29] Halpern JH, Pope HG Jr., Sherwood AR, Barry S, Hudson JI, Yurgelun-Todd D (2004). Residual neuropsychological effects of illicit 3,4-methylenedioxymethamphetamine (MDMA) in individuals with minimal exposure to other drugs. Drug and Alcohol Dependence 75, 135–147. 15276218 10.1016/j.drugalcdep.2004.02.008

[ref30] Halpern JH, Sherwood AR, Hudson JI, Gruber S, Kozin D, Pope HG Jr. (2011). Residual neurocognitive features of long-term ecstasy users with minimal exposure to other drugs. Addiction 106, 777–786. 21205042 10.1111/j.1360-0443.2010.03252.x PMC3053129

[ref31] Hanson KL, Luciana M (2004). Neurocognitive function in users of MDMA: the importance of clinically significant patterns of use. Psychological Medicine 34, 229–246. 14982129 10.1017/s0033291703001132

[ref32] Heffernan TM, Jarvis H, Rodgers J, Scholey AB, Ling J (2001). Prospective memory, everyday cognitive failure and central executive function in recreational users of ecstasy. Human Psychopharmacology: Clinical and Experimental 16, 607–612. 12404540 10.1002/hup.349

[ref33] Higgins JPT, Green S (editors) (2011). *Cochrane Handbook for Systematic Reviews of Intervention*, version 5.1.0. The Cochrane Collaboration (http://www.cochrane-handbook.org). Accessed February 2016.

[ref34] Hoshi R, Mullins K, Boundy C, Brignell C, Piccini P, Curran HV (2007). Neurocognitive function in current and ex-users of ecstasy in comparison to both matched polydrug-using controls and drug-naive controls. Psychopharmacology 194, 371–379. 17605005 10.1007/s00213-007-0837-5

[ref35] Jager G, de Win MML, van der Tweel I, Schilt T, Kahn RS, van den Brink W, van Ree JM, Ramsey NF (2008). Assessment of cognitive brain function in ecstasy users and contributions of other drugs of abuse: results from an fMRI study. Neuropsychopharmacology 33, 247–258. 17460617 10.1038/sj.npp.1301415

[ref36] Kish SJ, Lerch J, Furukawa Y, Tong J, McCluskey T, Wilkins D, Houle S, Meyer J, Mundo E, Wilson AA, Rusjan PM, Saint-Cyr JA, Guttman M, Collins DL, Shapiro C, Warsh JJ, Boileau I (2010). Decreased cerebral cortical serotonin transporter binding in ecstasy users: a positron emission tomography/[^11^C]DASB and structural brain imaging study. Brain 133, 1779–1797. 20483717 10.1093/brain/awq103 PMC2912692

[ref37] Lamers CTJ, Bechara A, Rizzo M, Ramaekers JG (2006). Cognitive function and mood in MDMA/THC users, THC users and non-drug using controls. Journal of Psychopharmacology 20, 302–311. 16510488 10.1177/0269881106059495

[ref38] McCann UD, Kuwabara H, Kumar A, Palermo M, Abbey R, Brasic J, Ye W, Alexander M, Dannals RF, Wong DF, Ricaurte GA (2008). Persistent cognitive and dopamine transporter deficits in abstinent methamphetamine users. Synapse 62, 91–100. 17992686 10.1002/syn.20471

[ref39] McCann UD, Peterson SC, Ricaurte GA (2007). The effect of catecholamine depletion by α-methyl-para-tyrosine on measures of cognitive performance and sleep in abstinent MDMA users. Neuropsychopharmacology 32, 1695–1706. 17203011 10.1038/sj.npp.1301302

[ref40] McCann UD, Szabo Z, Scheffel U, Dannals RF, Ricaurte GA (1998). Positron emission tomographic evidence of toxic effect of MDMA (“ecstasy”) on brain serotonin neurons in human beings. Lancet 352, 1433–1437. 9807990 10.1016/s0140-6736(98)04329-3

[ref41] McCann UD, Szabo Z, Seckin E, Rosenblatt P, Mathews WB, Ravert HT, Dannals RF, Ricaurte GA (2005). Quantitative PET studies of the serotonin transporter in MDMA users and controls using [^11^C]McN5652 and [^11^C]DASB. Neuropsychopharmacology 30, 1741–1750. 15841106 10.1038/sj.npp.1300736 PMC2034411

[ref42] McCardle K, Luebbers S, Carter JD, Croft RJ, Stough C (2004). Chronic MDMA (ecstasy) use, cognition and mood. Psychopharmacology 173, 434–439. 15088077 10.1007/s00213-004-1791-0

[ref43] Miyake A, Friedman NP (2012). The nature and organization of individual differences in executive functions: four general conclusions. Current Directions in Psychological Science 21, 8–14. 22773897 10.1177/0963721411429458 PMC3388901

[ref44] Miyake A, Friedman NP, Emerson MJ, Witzki AH, Howerter A, Wager TD (2000). The unity and diversity of executive functions and their contributions to complex “frontal lobe” tasks: a latent variable analysis. Cognitive Psychology 41, 49–100. 10945922 10.1006/cogp.1999.0734

[ref45] Moeller FG, Steinberg JL, Dougherty DM, Narayana PA, Kramer LA, Renshaw PF (2004). Functional MRI study of working memory in MDMA users. Psychopharmacology 177, 185–194. 15221201 10.1007/s00213-004-1908-5

[ref46] Molliver ME, Berger UV, Mamounas LA, Molliver DC, O'Hearn E, Wilson MA (1990). Neurotoxicity of MDMA and related compounds: anatomic studies. Annals of the New York Academy of Sciences 600, 640–661. 10.1111/j.1749-6632.1990.tb16916.x 1979216

[ref48] Monterosso JR, Aron AR, Cordova X, Xu J, London ED (2005). Deficits in response inhibition associated with chronic methamphetamine abuse. Drug and Alcohol Dependence 79, 273–277. 15967595 10.1016/j.drugalcdep.2005.02.002

[ref49] Montgomery C, Fisk JE (2008). Ecstasy-related deficits in the updating component of executive processes. Human Psychopharmacology: Clinical and Experimental 23, 495–511. 18512857 10.1002/hup.951

[ref50] Montgomery C, Fisk JE, Newcombe R, Murphy PN (2005 *a*). The differential effects of ecstasy/polydrug use on executive components: shifting, inhibition, updating and access to semantic memory. Psychopharmacology 182, 262–276. 16010539 10.1007/s00213-005-0065-9

[ref51] Montgomery C, Fisk JE, Newcombe R, Wareing M, Murphy PN (2005 *b*). Syllogistic reasoning performance in MDMA (ecstasy) users. Experimental and Clinical Psychopharmacology 13, 137–145. 15943546 10.1037/1064-1297.13.2.137

[ref52] Montgomery C, Fisk JE, Wareing M, Murphy PN (2007). Self reported sleep quality and cognitive performance in ecstasy users. Human Psychopharmacology: Clinical and Experimental 22, 537–548. 17960556 10.1002/hup.879

[ref53] Montgomery C, Hatton NP, Fisk JE, Ogden RS, Jansari A (2010). Assessing the functional significance of ecstasy-related memory deficits using a virtual reality paradigm. Human Psychopharmacology 25, 318–325. 20521322 10.1002/hup.1119

[ref54] Morgan MJ (1998). Recreational use of “ecstasy” (MDMA) is associated with elevated impulsivity. Neuropsychopharmacology 19, 252–264. 9718589 10.1016/S0893-133X(98)00012-8

[ref55] Morgan MJ, McFie L, Fleetwod LH, Robinson JA (2002). Ecstasy (MDMA): are the psychological problems associated with its use reversed by prolonged abstinence? Psychopharmacology 159, 294–303. 11862362 10.1007/s002130100907

[ref56] Murphy PN, Bruno R, Ryland I, Wareing M, Fisk JE, Montgomery C, Hilton J (2012). The effects of ‘ecstasy’ (MDMA) on visuospatial memory performance: findings from a systematic review with meta-analysis. Human Psychopharmacology: Clinical and Experimental 27, 113–138. 22389076 10.1002/hup.1270

[ref57] Murphy PN, Erwin PG, Maciver L, Fisk JE, Larkin D, Wareing M, Montgomery C, Hilton J, Tames FJ, Bradley B, Yanulevitch K, Ralley R (2011). The relationships of ‘ecstasy’ (MDMA) and cannabis use to impaired executive inhibition and access to semantic long-term memory. Human Psychopharmacology: Clinical and Experimental 26, 460–469. 21898599 10.1002/hup.1228

[ref58] Murphy PN, Wareing M, Fisk JE, Montgomery C (2009). Executive working memory deficits in abstinent ecstasy/MDMA users: a critical review. Neuropsychobiology 60, 159–175. 19893333 10.1159/000253552

[ref59] Nulsen C, Fox A, Hammond G (2011). Electrophysiological indices of altered working memory processes in long-term ecstasy users. Human Psychopharmacology: Clinical and Experimental 26, 488–497. 21953632 10.1002/hup.1231

[ref60] Nutt DJ, King LA, Phillips LD (2010). Drug harms in the UK: a multicriteria decision analysis. Lancet 376, 1558–1565. 21036393 10.1016/S0140-6736(10)61462-6

[ref61] Parrott AC (2009). Cortisol and 3,4-methylenedioxymethamphetamine: neurohormonal aspects of bioenergetic stress in ecstasy users. Neuropsychobiology 60, 148–158. 19893332 10.1159/000253551 PMC2826870

[ref62] Parrott AC (2013 *a*). Human psychobiology of MDMA or ‘ecstasy’: an overview of 25 years of empirical research. Human Psychopharmacology 28, 289–307. 23881877 10.1002/hup.2318

[ref63] Parrott AC (2013 *b*). MDMA, serotonergic neurotoxicity, and the diverse functional deficits of recreational ‘ecstasy’ users. Neuroscience and Biobehavioural Reviews 37, 1466–1484. 10.1016/j.neubiorev.2013.04.016 23660456

[ref65] Pazos A, Prosbit A, Palacios JM (1987). Serotonin receptors in the human brain – III. Autoradiographic mapping of serotonin-1 receptors. Neuroscience 21, 97–122. 2955249 10.1016/0306-4522(87)90326-5

[ref66] Ravizza SM, Ciranni A (2002). Contributions of the prefrontal cortex and basal ganglia to set shifting. Journal of Cognitive Neuroscience 14, 472–483. 11970806 10.1162/089892902317361985

[ref67] Reay JL, Hamilton C, Kennedy DO, Scholey AB (2006). MDMA polydrug users show process-specific central executive impairments coupled with impaired social and emotional judgement processes. Journal of Psychopharmacology 20, 385–388. 16574712 10.1177/0269881106063269

[ref68] Reneman L, Schilt T, de Win MM, Booij J, Schmand B, van den Brink W, Bakker O (2006). Memory function and serotonin transporter promoter gene polymorphism in ecstasy (MDMA) users. Journal of Psychopharmacology 20, 389–399. 16574713 10.1177/0269881106063266

[ref69] Ricaurte GA, DeLanney LE, Irwin I, Langston JW (1988). Toxic effects of MDMA on central serotonergic neurons in the primate: importance of route and frequency of drug administration. Brain Research 446, 165–168. 2897228 10.1016/0006-8993(88)91309-1

[ref70] Roberts CA, Fairclough S, Fisk JE, Tames FT, Montgomery C (2013 *a*). Electrophysiological indices of response inhibition in human polydrug users. Journal of Psychopharmacology 27, 779–789. 23803689 10.1177/0269881113492899

[ref71] Roberts CA, Fairclough SH, Fisk JE, Tames F, Montgomery C (2013 *b*). ERP evidence suggests executive dysfunction in ecstasy polydrug users. Psychopharmacology 228, 375–388. 23532375 10.1007/s00213-013-3044-6

[ref72] Roberts CA, Fairclough SH, McGlone FP, Fisk JE, Montgomery C (2013 *c*). Electrophysiological evidence of atypical processing underlying mental set shifting in ecstasy polydrug users. Experimental and Clinical Psychopharmacology 21, 507–515. 24080019 10.1037/a0034002

[ref73] Roberts CA, Montgomery C (2015 *a*). fNIRS suggests increased effort during executive access in ecstasy polydrug users. Psychopharmacology 232, 1571–1582. 25391436 10.1007/s00213-014-3795-8

[ref74] Roberts CA, Montgomery C (2015 *b*). Cortical oxygenation suggests increased effort during cognitive inhibition in ecstasy polydrug users. Journal of Psychopharmacology 29, 1170–1181. 26333432 10.1177/0269881115598412

[ref75] Roberts GMP, Garavan H (2010). Evidence of increased activation underlying cognitive control in ecstasy and cannabis users. NeuroImage 52, 429–435. 20417713 10.1016/j.neuroimage.2010.04.192

[ref76] Rodgers J (2000). Cognitive performance amongst recreational users of ‘ecstasy’. Psychopharmacology 151, 19–24. 10958112 10.1007/s002130000467

[ref77] Schilt T, de Win MML, Jager G, Koeter MW, Ramsey NF, Schmand B, van den Brink W (2008). Specific effects of ecstasy and other drugs on cognition in poly-substance users. Psychological Medicine 38, 1309–1317. 17988417 10.1017/S0033291707002140

[ref78] Semple DM, Ebmeier KP, Glabus MF, O'Carrol RE, Johnstone EC (1999). Reduced *in vivo* binding to the serotonin transporter in the cerebral cortex of MDMA (‘ecstasy’) users. British Journal of Psychiatry 175, 63–69. 10621770 10.1192/bjp.175.1.63

[ref79] Sterne JAC, Sutton AJ, Ioannidis JPA, Terrin N, Jones JR, Lau J, Carpenter J, Rücker G, Harbord RM, Schmid CH, Tetzlaff J, Deeks JJ, Peters J, Macaskill P, Schwarzer G, Duval S, Altman DG, Moher D, Higgins JPT (2011). Recommendations for examining and interpreting funnel plot asymmetry in meta-analyses of randomised controlled trials. British Medical Journal 343, d4002. 21784880 10.1136/bmj.d4002

[ref80] Stuss DT, Alexander MP, Hamer L, Palumbo C, Dempster R, Binns M, Levine B, Izukava D (1998). The effects of focal anterior and posterior brain lesions on verbal fluency. Journal of the International Neuropsychological Society 4, 265–278. 9623001

[ref81] Thomasius R, Petersen K, Bucherts R, Andresen B, Zapletalova P, Wartberg L, Nebeling B, Schmoldt A (2003). Mood, cognition and serotonin transporter availability in current and former ecstasy (MDMA) users. Psychopharmacology 167, 85–96. 12632248 10.1007/s00213-002-1383-9

[ref82] Urban NBL, Girgis RR, Talbot PS, Kegeles LS, Xu X, Frankle WG, Hart CL, Slifstein M, Abi-Dargham A, Laruelle M (2012). Sustained recreational use of ecstasy is associated with altered pre and post synaptic markers of serotonin transmission in neocortical areas: a PET study with [^11^C]DASB and [^11^C]MDL 100907. Neuropsychopharmacology 37, 1465–1473. 22353758 10.1038/npp.2011.332 PMC3327851

[ref83] von Geusau NA, Stalenhoef P, Huizinga M, Snel J, Ridderinkhof KR (2004). Impaired executive function in male MDMA (‘ecstasy’) users. Psychopharmacology 175, 331–341. 15034712 10.1007/s00213-004-1832-8

[ref84] Wareing M, Fisk JE, Montgomery C, Murphy PN, Chandler MD (2007). Information processing speed in ecstasy (MDMA) users. Human Psychopharmacology 22, 81–88. 17295197 10.1002/hup.827

[ref85] Wareing M, Fisk JE, Murphy PN, Montgomery C (2004). Verbal working memory deficits in current and previous users of MDMA. Human Psychopharmacology 19, 225–234. 15181650 10.1002/hup.586

[ref86] Wareing M, Fisk JE, Murphy PN, Montgomery C (2005). Visuo-spatial working memory deficits in current and former users of MDMA (‘ecstasy’). Human Psychopharmacology 20, 115–123. 15641126 10.1002/hup.670

[ref87] Wetherell MA, Montgomery C (2014). Basal functioning of the hypothalamic–pituitary–adrenal (HPA) axis and psychological distress in recreational ecstasy polydrug users. Psychopharmacology 231, 1365–1375. 24190587 10.1007/s00213-013-3325-0

[ref88] Winstock A (2015). The Global Drugs Survey 2015 (http://www.globaldrugsurvey.com/the-global-drug-survey-2015-findings/). Accessed February 2016.

[ref89] Yip JTH, Lee TMC (2005). Effect of ecstasy use on neurophysiological function. Psychopharmacology 179, 620–628. 15650845 10.1007/s00213-004-2083-4

[ref90] Zakzanis KK, Young DA (2001). Executive function in abstinent MDMA (‘ecstasy’) users. Medical Science Monitor: International Medical Journal of Experimental and Clinical Research 7, 1292–1298. 11687745

