# Supplementary material for: Meta-analysis of executive functioning in ecstasy/polydrug users
Source: Psychol Med. 2016 Mar 11;46(8):1581–96. doi: 10.1017/S0033291716000258 (PMC4873937; doi:10.1017/S0033291716000258)
Supplement: Supplementary file 1 [file S0033291716000258sup001.doc]

***Supplementary Table S1.*** *Summary of studies included in meta-analysis on executive function in current ecstasy users and drug-*using controls

| **Authors and Study** | **Participants and Design** | **Task(s) used, outcome (dv in anlysis) and function assessed** | **Previous exposure to other drugs** | **Result** |
| --- | --- | --- | --- | --- |
| Bedi & Redman (2008) | 45 ecstasy polydrug users (47% F, mean age: 22.8±3.0, MLD = 170.6±362.8, MTSLU = 79.2±108.5 days)  48 Cannabis polydrug users (46% F, mean age = 21.7±3.5)  Other groups not included in meta-analysis = 40 legal drug users. | COWA FAS – total words (access) | Ecstasy polydrug users and cannabis polydrug users report use of alcohol, nicotine, amphetamine, nitrous oxide, LSD, magic mushrooms, cocaine, Amyl nitrate, benzodiazepines and cannabis.  Ecstasy users were also exposed to ketamine. | No between group differences in original analysis |
| Croft et al. (2001) | 11 MDMA and cannabis users (55% F, mean age: 27.5±4.7, MLD = 41.9 (49.3), no ecstasy abstinence data given)  18 cannabis users (22% F, mean age: 26.6±8.1)  Other groups not included in meta-analysis = 31 drug naïve controls | COWA FAS – total words (access)  Stroop – interference RT (inhibition)  Digit span backwards – task score (updating) | Both drug user groups report use of cannabis, cocaine, speed and alcohol. | No differences in performance between MDMA users and cannabis users, however there were differences between a combined drug user group and drug naïve controls in digit span backwards performance (drug naïve controls performed better) |
| Dafters (2006a) | 33 ecstasy and cannabis users (36% F, mean age: 23.09±2.34, MLD = 499.1±671.56, min abstinence = 5 days)  18 non-users (44% F, mean age: 22.67)  Other groups not included in meta-analysis = 17 cannabis users (not included due to ecstasy use being relatively high in this group for a control group) | Stroop – interference RT (inhibition)  Stroop switch – switch RT (switching)  Keep track – total words (updating) | Non-users exposed to alcohol, tobacco and amphetamine. Ecstasy-cannabis users report exposure to cannabis, cocaine, amphetamine, alcohol, tobacco, heroin and LSD. | Ecstasy users, significantly impaired on task-switching Stroop, but not in Stroop interference or Keep Track Task. |
| Dafters (2006b) | 18 ecstasy and cannabis users (33% F, mean age: 23.24±2.33, MLD = 522.33±936.71, min abstinence = 5 days)  18 non-users (44% F, mean age: 22.67±2.56)  Other groups not included in meta-analysis = 18 cannabis users (not included due to ecstasy use being relatively high in this group for a control group) | Stroop – interference RT (inhibition) | Non-users exposed to alcohol, tobacco and amphetamine. Ecstasy-cannabis users report exposure to cannabis, cocaine, amphetamine, alcohol, tobacco, heroin and LSD. | No significant between group differences |
| de Sola et al. (2008a) | 37 ecstasy polydrug users (49% F, mean age: 23.6±3.5, MLD = 206±228.3, min abstinence = 72h)  23 cannabis users (65% F, mean age: 22.0±1.9)  Other groups not included in meta-analysis = 34 drug naïve controls | ToL – total movements (switching) | The ecstasy polydrug user group and the cannabis user group both had previous exposure to alcohol, tobacco and cannabis.  Drug screens conducted with urine and hair screens. | No significant between group differences at baseline |
| de Sola et al. (2008b) | 14 ecstasy polydrug users (57% F, mean age = 25.2±3.3, MLD = 207.4±151.0, no abstinence data given)  13 cannabis users (61% F, mean age: 25.1±2.9)  Other groups not included in meta-analysis = 22 drug naïve controls | ToL – total movements (switching) | The ecstasy polydrug user group and the cannabis user group both had previous exposure to alcohol, tobacco and cannabis.  Drug screens conducted with urine and hair screens. | No significant between group differences at baseline |
| Fisk & Montgomery (2009) | 14 heavy ecstasy users (36% F, mean age: 22.86, MLD = 1000.21±786.41, MTSLU = 22 weeks)  28 non-users (75% F, mean age: 20.71)  Other groups not included in meta-analysis = 39 light ecstasy users | RLG – task score (inhibition)  Computation span - % cost (updating)  Consonant updating – task score (updating)  Spatial updating - task score (updating) | Non-users report exposure to alcohol, tobacco and cannabis. In addition to these drugs, ecstasy users also report cocaine use. | Heavy user not impaired at RLG . All updating measures show ecstasy related deficits, and these were significant in 2 out of 3 measures. |
| Fisk et al. (2004) | 44 ecstasy users (mean age: 21.52±1.66, MLD = 343.38±376.94, MTSLU = 10.90±27.86 weeks)  59 non-users (mean age: 21.37±1.84) | RLG – task score (inhibition)  Computation span – task score (updating) | Both groups report exposure to alcohol, tobacco, cannabis, cocaine and amphetamine. | No group differences on RLG performance. Ecstasy users significantly impaired on computation span |
| Fox et al. (2001) | 11 high intensity ecstasy users (45% F, mean age: 28.0±5.3, MTSLU = 2.8±5.9 months)  20 polydrug controls (70% F, mean age: 23.3±6.5)  Other groups not included in meta-analysis = 14 low and 14 medium intensity ecstasy users | WCST - % perseverative errors (switching)  ToL – solution time (switching) | High intensity ecstasy users and polydrug controls were exposed to cannabis, amphetamine, cocaine, LSD, barbiturates, opiates, psilocybin mushrooms, nicotine and alcohol. | No between group differences in WCST perseverative errors or ToL solution time |
| Fox et al. (2002) | 20 ecstasy polydrug users (50% F, mean age: 27.3±6.7, MLD = 172.0±227.36, MTSLU = 51.9±25.9 months)  20 polydrug controls (60% F, mean age: 27.5±7.6) | 3D IDED – latency (switching) | Ecstasy users and controls report exposure to cannabis, amphetamine, cocaine, LSD, psilocybin mushrooms, opiates, solvents, nicotine and alcohol. | No between group differences |
| Gouzoulis-Mayfrank et al. (2000) | 28 ecstasy users (43% F, mean age: 23.25, MDL = 93.4±119.9, MTSLU = 41±71.1 days)  28 polydrug controls (46% F, mean age: 22.9)  Other groups not included in meta-analysis = 28 drug naïve controls | Stroop – interference factor (inhibition)  Digit span backwards – task score (updating)  Phonological word fluency – total words (access) | Ecstasy users and polydrug controls report previous exposure to cannabis, alcohol and nicotine.  Drug screens performed by urine samples. | Ecstasy users performed worse than non-users in digit span backwards. No performance differences observed in Stroop interference or word fluency |
| Gouzoulis-Mayfrank et al. (2003) | 30 heavy ecstasy users (30% F, mean age: 25.1±4.65, MDL = 503.2±555.5, MTSLU = 194.8±351.8 days)  30 non-users (30% F, mean age: 25.37±2.72)  Other groups not included in meta-analysis = 30 moderate ecstasy users | Go NoGo – correct responses (inhibition)  Digit span backwards – task score (updating)  2back letters – correct responses (updating)  2back figures – correct responses (updating) | Non-users report mild cannabis use, alcohol use and tobacco use.  Heavy ecstasy users report previous exposure to cannabis, alcohol, tobacco, amphetamine and cocaine. | No differences between ecstasy users and controls in central executive function. |
| Halpern et al. (2004) | 23 ecstasy users with minimal exposure to other drugs (65% F, mean age: 20, MDL = 60 episodes)  16 controls equally involved in rave culture (44% F, mean age: 22) | COWA FAS – total words (access)  Stroop – interference RT (inhibition)  WCST - total perseverative errors (switching)  Digit span backwards – task score (updating) | Exclusion criteria for both groups: lifetime episodes of cannabis use >50. More than 4 episodes of sedative-hypnotics,  opioids, cocaine, stimulants, hallucinogens, phencyclidine,  ketamine, gamma-hydroxybutyrate (GHB), or inhalants, would lead to exclusion.  Both groups report exposure to alcohol and tobacco. | No between group differences in FAS, WCST, Stroop or digit span backwards. However ecstasy related impairment on digit span backwards when adjusted for age and sex |
| Halpern et al. (2011) | 52 ecstasy users (46% F, mean age: 22, MDL = 43.5 episodes, MTSLU = 121 days)  59 non-users (36% F, mean age: 24) | Spatial span backwards - task score (updating)  Digit span backwards – task score (updating)  Stroop – interference RT (inhibition)  WCST - total perseverative errors (switching)  TMT-B – time (switching) | Participants were excluded from both groups if their reported more than 100 lifetime  episodes of cannabis use or more than 10 exposures to any other class of illicit drugs. | No significant between group differences on any of the executive measures. |
| Hanson & Luciana (2004) | 26 ecstasy users (46% F, mean age: 21.3±3.6, MLD = 123.31, MTSLU = 10.9±10.5 weeks)  26 non-users (46% F, mean age: 20.7±3.4) | COWA FAS – total words (access)  Digit span backwards – task score (updating) | Use of drugs other than ecstasy was permitted for both groups. Previous exposure not reported in the paper. | No between group differences in COWA total words, or digit span backwards performance |
| Heffernan et al. (2001) | 30 regular ecstasy users (43% F, mean age: 23.9±4.47, min TSLU = 24h)  37 ecstasy free controls (73% F, mean age: 25.5±8.76) | Word fluency, C letter words – total words (access) | Ecstasy users had previous exposure to cannabis, cocaine and alcohol  There was cannabis exposure and alcohol exposure in the control group. | Ecstasy users performed significantly worse than controls in verbal fluency measure. |
| Hoshi et al. (2007) | 25 ecstasy users (mean age: 28.64±4.59, MLD = 1111.68, MTSLU = 14.2 days)  29 polydrug users (mean age: 31.93±8.41)  Other groups not included in meta-analysis = 28 ex-ecstasy users and 27 drug naïve controls | Subtracting serial sevens – errors (updating)  Verbal fluency – total words (access)  TMT-B – time (switching)  Go Nogo – errors (inhibition) | Ecstasy users and polydrug users had a range of exposure to other recreational drugs including cocaine, cannabis, amphetamines and LSD.  Drug use quantified by hair samples. | No significant group differences were found in Serial Sevens, verbal fluency, the TMT.  Polydrug users had more errors than both ex-users and controls on Go NoGo, but were not significantly different to current users |
| Lamers et al. (2006) | 11 MDMA/THC users (mean age: 22.9±2.4, MTSLU = 228.1±140.3 days)  15 cannabis users (mean age: 24.3±5.3)  Other groups not included in meta-analysis = 15 non-drug users | TMT-B – time (switching)  WCST - total perseverative errors (switching) | Both drug user groups report previous exposure to cannabis, alcohol and nicotine.  Urine drug screen performed. | No between group effects on TMT-B or WCST |
| McCardle et al. (2004) | 17 ecstasy users (24% F, mean age: 21.06±1.56), MTSLU = 130 days)  15 controls (13% F, mean age: 21.91±1.62) | Digit span backwards – task score (updating)  TMT-B – time (switching) | Ecstasy users and controls, both report exposure to cannabis, hallucinogens, amphetamine, alcohol, other opiates, tranquilizers, barbiturates, tobacco, heroin, amyl nitrate, cocaine and other inhalants. | No between group effects observed in digit span backwards or TMT-B |
| Montgomery & Fisk (2008) | 73 ecstasy polydrug (47% F, mean age: 21.77±2.11, MLD = 309.86±486.25, MTSLU = 32.15±62.82 weeks)  73 non-ecstasy users (73% F, mean age: 20.73±1.73) | Letter updating – task score (updating)  Spatial updating – task score (updating) | Ecstasy users and controls report previous exposure to cannabis, cocaine and alcohol. | Ecstasy users impaired in four out of six sub-sample analyses. |
| Montgomery et al. (2005a) | Study 1: 27 ecstasy users (48% F, mean age: 21.70±1.66, MLD = 345.96±365.76, MTSLU = 4.97±7.27 weeks)  34 non-users (71% F, mean age: 21.59±1.88)  Study 2: 51 ecstasy users (47% F, mean age: 21.96±2.11, MLD = 373.87±542.91, MTSLU = 22.15 weeks)  42 non-users (79% F, mean age: 20.83±1.45) | CWFT C letter words – total words (access)  Computation span – task score (updating)  Letter updating – task score (updating)  Number-letter task – switch cost (switching)  Plus-minus task – switch cost (switching)  RLG – task score (inhibition) | Use of other drugs was limited to cannabis, alcohol and tobacco among the non-ecstasy group in both studies. However ecstasy users also report cocaine and amphetamine use. | Ecstasy users performed worse on both updating tasks and access to  long-term memory tasks.  Ecstasy users performed significantly  better on the inhibition task. No group differences were observed in switching. |
| Montgomery et al. (2005b) | 22 MDMA users (50% F, mean age: 21.36±1.67, MLD = 303.3±374.04, MTSLU = 4.61±6.82 weeks)  26 non-MDMA users (62% F, mean age: 21.31±1.69) | RLG – task score (inhibition)  Computation span – task score (updating) | Use of other drugs  was common in MDMA users, whereas among nonusers, drug use was mainly limited to alcohol,  cannabis, and tobacco | Ecstasy users performed significantly worse than non-users in the computation span task. There were no group differences in RLG performance |
| Montgomery et al. (2007) | 104 ecstasy users (mean age: 21.68±1.96, MLD = 349.97±464.41, MTSLU = 19.35±43.46 weeks)  103 non-users (mean age: 21.11±1.66) | CWFT – standardised score (access)  Computation span - % cost (updating)  Letter updating – task score (updating) | Use of other drugs  was common in MDMA users, whereas among nonusers, drug use was mainly limited to alcohol,  cannabis, and tobacco | Ecstasy users performed worse than controls on all measures |
| Morgan (1998) | Study 1: 16 ecstasy users (50% F, mean age: 20.94±1.88, MLD = 35.5±17.5, MTSLU = 20.4±33.6 days)  12 polydrug controls (mean age: 20.25±1.48)  Other groups not included in meta-analysis = 16 non-drug controls  Study 2: 25 ecstasy users (52% F, mean age: 22.28±2.48, MLD = 49.6±33.2, MTSLU = 65.1±85.7)  20 polydrug controls (mean age: 23±4.71)  Other groups not included in meta-analysis = 16 non-drug controls | ToL – proportion of perfect solutions (switching) | Ecstasy users and polydrug controls report previous exposure to alcohol, nicotine, amphetamine, cannabis, LSD, inhalants, cocaine and psilocybin mushrooms. | No between group differences of ToL performance in either study |
| Morgan et al. (2002) | 18 ecstasy users (50% F, mean age: 23.4±3.2, MLD = 303±267.5, MTSLU = 4.05±3.2 weeks)  16 polydrug users (50% F, mean age: 22.1±3.3)  Other groups not included in meta-analysis = 15 ex-ecstasy users, 15 drug naïve controls | TMT-B – time (switching)  COWA FAS – total words (access)  Stroop – interference RT (inhibition)  Subtracting serial sevens – errors (updating) | Both groups were permitted use of other drugs. There was similar exposure to cannabis, amphetamine, cocaine, LSD, psilocybin mushrooms and poppers in each group. | Ecstasy users worse on SSS than all groups. However, no between group differences observed in verbal fluency, Stroop interference RT, or TMT-B completion time. |
| Murphy et al. (2011) | 15 ecstasy and cannabis users (73% F, mean age: 24.5±3.4, MLD = 364.8±665.1, MTSLU = 365 days)  13 cannabis users (54% F, mean age: 21.9±4.6)  Other groups not included in meta-analysis = 12 drug naïve controls | RLG – task score (inhibition) | Ecstasy users had exposure to cannabis, cocaine and amphetamine.  Cannabis group had exposure to cannabis and minimal exposure to cocaine and amphetamines. | Ecstasy users had significantly higher redundancy on RLG than drug naïve controls but not cannabis controls  Cannabis use predicted redundancy in a regression model |
| Nulsen et al. (2011) | 11 ecstasy users (64% F, mean age: 22.9±2.6, MLD = 32.5±27.2)  13 polydrug controls (70% F, mean age: 23.2±3.3)  Other groups not included in meta-analysis = 13 drug naïve controls | Digit span backwards – task score (updating) | Ecstasy users and polydrug users were exposed to cannabis, LSD and cocaine.  Saliva samples were used to detect THC. | No significant between group differences in digit span backwards performance |
| Reay et al. (2006**)** | 15 ecstasy polydrug users (40% F, mean age: 25±5.8, MLD = 593.4)  15 polydrug controls (53% F, mean age = 21.3±538) | Digit span backwards – task score (updating)  Brixton spatial anticipation task – errors (switching)  Inhibition of return – mean slowing (s) (inhibition) | Polydrug controls report recent cannabis, alcohol and tobacco consumption. Ecstasy users also had recent concaine consumption. | Ecstasy users performed significantly worse on digit span backwards and the Brixton spatial anticipation task. No between group differences observed in inhibition of return |
| Reneman et al. (2006) | 23 heavy ecstasy (48% F, mean age: 26.05±5.05, MLD = 516.35±452.1, MTSLU = 2.29±2.39 months)  15 polydrug controls (53% F, mean age: 26.3±4.1)  Other groups not included in meta-analysis =15 moderate MDMA users, 16 ex MDMA users | COWA FAS – total words (access)  Stroop – interference RT (inhibition)  WCST - total perseverative errors (switching)  TMT-B – time (switching) | Polydrug controls report previous exposure to alcohol, tobacco and cannabis.  Ecstasy users report previous exposure to alcohol, tobacco, cannabis, amphetamine and cocaine.  Drug screens were carried out using urine samples. | No between group differences overall on executive functioning |
| Roberts et al. (2013a) | 20 ecstasy polydrug users (50% F, mean age: 23.95±2.50, MLD = 177.65±301.73, min abstinence = 7 days)  20 polydrug controls (55% F, mean age = 22.58±3.45)  Other groups not included in meta-analysis = 20 drug naïve controls | Go NoGo – NoGo errors (inhibition) | Ecstasy polydrug users and polydrug user groups have exposure to cannabis, cocaine and ketamine.  Drug screen conducted by urine analysis. | No between group differences in NoGo errors |
| Roberts et al. (2013b) | 20 ecstasy polydrug users (50% F, mean age: 23.95±2.50, MLD = 177.65±301.73, min abstinence = 7 days)  20 polydrug controls (55% F, mean age = 22.58±3.45)  Other groups not included in meta-analysis = 20 drug naïve controls | Semantic retrieval task – low association errors (access) | Ecstasy polydrug users and polydrug user groups have exposure to cannabis, cocaine and ketamine.  Drug screen conducted by urine analysis. | No behavioural between group differences |
| Roberts et al. (2013c) | 20 ecstasy polydrug users (50% F, mean age: 23.95±2.50, MLD = 177.65±301.73, min abstinence = 7 days)  20 polydrug controls (55% F, mean age = 22.58±3.45)  Other groups not included in meta-analysis = 20 drug naïve controls | Number-letter task – switch cost (switching) | Ecstasy polydrug users and polydrug user groups have exposure to cannabis, cocaine and ketamine.  Drug screen conducted by urine analysis. | No behavioural between group differences |
| Rodgers (2000) | 15 ecstasy users (53% F, mean age: 31 years 5 months, MLD = 20 occasions, min abstinence = 2 months)  15 cannabis users (53% F, mean age = 30 years 3 months)  Other groups not included in meta-analysis = 15 drug naïve controls | Digit span – task score (updating) | Ecstasy users report use of cannabis, LSD, amphetamine and cocaine.  The cannabis user group were exposed to cannabis only. | No performance difference in digit span |
| von Geusau et al. (2004) | 26 ecstasy users (35% F, mean age: 21.55±1.3, min abstinenece = 2 weeks )  33 non-users (64% F, mean age: 21.7±2.1) | WCST - total perseverative errors (switching)  ToL – total movements (switching)  Stop signal task – Stop signal RT (inhibition)  Mental counters - % correct (updating) | Ecstasy user group showed exposure to cannabis, LSD, amphetamine, psilocybin, cocaine, GHB, sedative and herbal E.  Controls report low levels of exposure to cannabis, psilocybin and sedatives. | Male MDMA users performed worse on tasks that tap cognitive flexibility. No differences were observed on other cognitive tasks. Female users showed no impairments |
| Wareing et al. (2004) | 42 ecstasy users (48% F, mean age: 21.69±2.57, MLD = 552.99±681.41, MTSLU = 3±3.66 weeks)  31 non-users (61% F, mean age: 23.39±6.47) | Computation span – task score (updating) | MDMA users report cannabis use, amphetamine and poppers.  A third of non-users report exposure to cannabis. | MDMA users performed significantly worse than controls on computation span task |
| Wareing et al. (2005) | 36 ecstasy users (mean age: 21.81, MLD = 591.33±718.44, MTSLU = 3.30±3.87)  31 non-users (mean age: 23.39±6.47)  Other groups not included in meta-analysis =12 former ecstasy users | Spatial working memory span – score (updating)  Computation span – task score (updating) | Ecstasy users and non-users both report a history of alcohol, amphetamine, cannabis, cocaine, mushrooms, poppers and tobacco use. | Ecstasy users (users and former users) show impaired spatial working memory compared to controls. |
| Wareing et al. (2007) | 29 ecstasy users (mean age: 21.72±2.00, MLD = 536±515.73, MTSLU = 1.86±1.50 weeks)  46 non-users (mean age: 22.85±5.50)  Other groups not included in meta-analysis = 10 previous users | Computation span – task score (updating) | MDMA users report cannabis use, amphetamine and poppers.  A third of non-users report exposure to cannabis. | Both ecstasy user groups performed significantly worse than non-users on the computation span measure |
| Yip & Lee (2005) | 100 ecstasy users (mean age: 28.48±5.71, MLD = 35.81±13.21, MTSLU = 2.23±0.51 months)  100 non-users (mean age: 28.82±5.78) | Stroop – interference RT (inhibition)  Digit span backwards – task score (updating) | Neither group had exposure to illicit substances other than ecstasy | No between group differences on backwards digit span. However ecstasy users performed significantly worse at the Stroop task |
| Zakzanis & Young (2001) | 30 ecstasy users (67% F, mean age: 22.96, MLD = 37.76, MTSLU = 19.96 weeks)  24 non-users (67% F, mean age: 19.54) | Rule shift cards test – errors (switching) | In the MDMA group use of amphetamines, cocaine, sedatives, LSD, cannabis and PCP was reported.  There were reports of cannabis use, amphetamines, sedatives and LSD in the non-user group  Abstinence confirmed by urine and blood samples. | No significant difference between groups in rule shift cards test performance |
